# Predicting Chinese Adolescent Internet Gaming Addiction From Peer Context and Normative Beliefs About Aggression: A 2-Year Longitudinal Study

**DOI:** 10.3389/fpsyg.2018.01143

**Published:** 2018-07-06

**Authors:** Ping Su, Chengfu Yu, Wei Zhang, Sha Liu, Yang Xu, Shuangju Zhen

**Affiliations:** ^1^School of Psychology, South China Normal University, Guangzhou, China; ^2^School of Education, Guangzhou University, Guangzhou, China

**Keywords:** internet gaming addiction, peer victimization, deviant peer affiliation, normative beliefs about aggression, longitudinal study

## Abstract

There has been growing concern over Internet gaming addiction (IGA) around the world. However, the risk factors and mediating mechanisms of IGA in Chinese youth remain largely unknown. A total of 323 Chinese adolescents (52.94% females, *M* age = 14.83, *SD* = 0.49, range = 13.50–16.50) completed questionnaires regarding peer victimization, deviant peer affiliation (DPA), normative beliefs about aggression (NBA), and IGA in the fall semester of 7th, 8th, and 9th grade. Structural equation modeling showed that 7th grade peer victimization predicted higher 8th grade DPA, which in turn was associated with enhanced 9th grade NBA, and ultimately, higher 9th grade IGA. In addition, 7th grade peer victimization made a unique contribution to 9th grade IGA through 9th grade NBA. The current study goes beyond previous research by using a 2-year longitudinal design and by taking into account both peer relations and individual cognitions as predictors of IGA. In addition, these findings have practical significance for improving intervention strategies targeting risk factors for adolescent IGA.

## Introduction

During the past decade, the online game industry has grown to $93 billion world-wide (Gartner, 2013). By early 2017, the scale of Chinese Internet gaming players reached 417 million, with the majority (57%) being between 10 and 25 years old. In addition, the rate of excessive Internet dependence has been estimated to be 64.3% (CNNIC, 2017). In the Chinese context, the topic of Internet gaming addiction (IGA) needs further attention and further research (Griffiths et al., 2015). IGA is a subtype of problematic Internet use (Block, 2008), which can be defined as uncontrollable, excessive, and compulsive use of online games that causes social and/or emotional problems (Young and de Abreu, 2011). Many empirical studies indicate that IGA is closely associated with adolescents’ cognitive, social, and/or physical maladjustment (Yu et al., 2013; Granic et al., 2014; Lobel et al., 2014), such as symptoms of generalized anxiety disorder, social phobia, insomnia, and hormonal disorders (Allison et al., 2006).

It is particularly important to understand Chinese youth’s IGA in the Chinese cultural context (Cao and Su, 2007; Funk, 2007; Wang et al., 2011). China’s current conditions are extremely different from those of other developed countries, so that many Chinese adolescents lack developmental support of the kind that might prevent addiction. Firstly, Chinese middle school students have to face so much great changes which taken place in both physical and mental fields, and the academic burden of them is getting heavier and heavier. But the Chinese Education Bureau focuses exclusively on academics in middle school, without much needed outdoor activities. Because of academic pressure, the majority of these Chinese students do not have enough time to enjoy extracurricular activities. Under stress, these students may turn to Internet gambling for release (Song and Qiao, 2010). A second cultural factor has to do with China’s “One Child Policy,” which was in effect from 1979 to early 2016. Most of today’s adolescents were only children without a family playmate. School has become the only place for adolescents to make friends, but it seems to be difficult to develop high-quality peer relationships with so little free time during and after school. It is possible that for some students, Internet gaming could be a way to maintain online friendships (Zhu et al., 2015). However, it is unknown whether conclusions based on studies conducted in other countries are applicable to Chinese adolescents’ IGA or not. Therefore, it is crucial to examine risk factors for IGA in the Chinese context.

### Peer Victimization, Deviant Peer Affiliation, and Adolescent IGA

The influence of peers is strengthened during adolescence (Plaisier and Konijn, 2013; Rudolph et al., 2014). Many negative aspects of the peer context might become even more negative during adolescence (Lopez and Dubois, 2005). Exposure to peer victimization is a salient stressor that affects numerous aspects of their growing up (Hanish and Guerra, 2002; Card and Hodges, 2008). Peer victimization refers to the suffering of repeated bullying behavior such as physical, verbal, or relational aggression or threats of aggression (Olweus, 1996). Frequently victimized youth may lose their social standing and be marked as outcasts (Bukowski and Sippola, 2001). Numerous studies have demonstrated that adolescents who were more often victimized were also more likely to indulge in online games (Zhou et al., 2014; Kim et al., 2015). The satisfaction theory of Internet addiction indicates that when the victimized individuals cannot get enough peer support in real life, they will play Internet games to try to meet psychological needs, thus causing excessive dependence on Internet games and even more difficulties with self-control themselves (Swickert et al., 2002; Young-Jones et al., 2014). Online gaming, and role playing games in particular, may compensate for the negative effects of peer victimization by helping the adolescent build new relationships and regain confidence (Morahan-Martin and Schumacher, 2003). It is reasonable to assume that peer victimization can increase the risk of IGA.

In addition to considering the direct relationship between peer victimization and IGA, we need to further consider possible mediating factors. Recent studies have shown that deviant peer affiliation (DPA) is a significant partial mediator in the relationship between perceived school climate (such as peer victimization) and adolescent IGA (Jiang et al., 2016; Li et al., 2016a,b). DPA means that adolescents make friends who themselves engaged in deviant behaviors, such as cheating, stealing, fighting and alcohol use. It is also well documented that DPA is intrinsically related to adolescent IGA (Ko et al., 2007; Li et al., 2015, 2016a; Wang et al., 2015; Zhu et al., 2015). Our first goal in the current study was to identify the pathway through which peer victimization would contribute directly to DPA, which could then predict adolescent IGA.

We draw from the social development model (Hawkins and Weis, 1985) and social network theory (Veenstra and Dijkstra, 2011) to conceptualize that the link between peer victimization and affiliation with deviant peers. According to these theories, exposure to peer victimization will undermine adolescents’ engagement with the mainstream peer group, who may mark them as outcasts. Given their low status and compromised friendships, victimized individuals may be willing to make friends with deviant peers who are perceived similarly as social outcasts, and may join the deviant group to compensate for social alienation (Bukowski and Sippola, 2001; Rudasill et al., 2013; Rudolph et al., 2014). Indeed, research suggests that victimization predicts lower acceptance and more rejection in the peer group (Kochel et al., 2012), as well as more difficulty forming new friendships (Ellis and Zarbatany, 2007). The social learning theory emphasized that in the process of social interaction with deviant peers, adolescents will engage in negative behaviors such as IGA through imitation and reinforcement (Dodge et al., 2006). Moreover, in the Chinese context, Internet bars provide a social context for playing online games, reinforcing these processes of imitation and reinforcement net bar together all day long (Zhu et al., 2015). Based on the literature reviewed above, we proposed the following hypothesis:

*Hypothesis 1* Deviant peer affiliation will mediate the association between peer victimization and adolescent IGA.

### Normative Beliefs About Aggression as a Mediator

Ecological systems theory (Bronfenbrenner, 1977) considers environmental factors across multiple systems as well as individuals’ involvement in the developmental process. More accurately, the updated bioecological theory pinpoints the critical role of individuals, specified as proximal influences, in the developmental process. In the current study, we focus on normative beliefs about aggression (NBA) as one of individual factors that might shape adolescents’ IGA. Normative beliefs serve to regulate actions by setting up the range of permitted and prohibited behaviors that are expected in a given culture (Huesmann and Guerra, 1997). Individuals who develop strong NBA adopt corresponding behaviors related to aggression. The Chinese Gaming Report (2012) sampled 117 popular Internet games and found that nearly 70% of the games involved violence, and NBA may facilitate the development of online gaming addiction (Cui et al., 2006; Choo et al., 2010; Gentile et al., 2011). Because there is the possibility of interpersonal misunderstandings in computer-mediated interactions, competition and violence-related clues could encourage adolescents to be aggressive (McKenna and Bargh, 2000; Ybarra and Mitchell, 2007).

On the basis of this understanding, the current study focused on NBA (a psychological factor) as a mediator in the relationship between peer context (environmental factor) and adolescent IGA. Peer victimization is assumed to increase the risk of NBA directly or indirectly by way of DPA. Firstly, the experience of victimization will motivate adolescents’ negative emotions and tendency toward revenge, thereby contributing to stronger acceptance of aggression beliefs (Sukhodolsky and Ruchkin, 2006). When playing online games, however, the player is empowered to fight and no longer be victimized (Krahé and Möller, 2004). Secondly, in deviant peer groups, peers’ normative beliefs supportive of aggression might have a considerable impact on the adolescent (Ang et al., 2011). Social learning theory argues that adolescents tend to adopt a view of aggression as normative by observing, imitating and reinforcing negative behavior in the process of social interaction (Svensson, 2003). Based on the above theoretical perspectives, we proposed the following hypothesis:

*Hypothesis 2* Normative beliefs about aggression will mediate the effect of peer victimization and DPA on adolescent IGA.

### The Present Study

**FIGURE 1:**
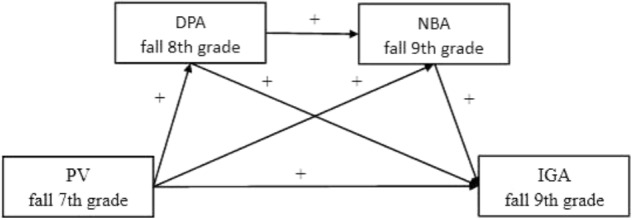
The proposed mediation model. PV, peer victimization; DPA, deviant peer affiliation; NBA, normative beliefs about aggression; IGA, internet gaming addiction.

In brief, the present study brings together two hypotheses to constitute a “serial mediation” model in which a sequence of two mediators explains the connection between a predictors and an outcome. **Figure 1** illustrates the full proposed research model. In this research, we used a 2-year longitudinal design to study how peer victimization in 7th grade was associated with IGA in 9th grade. We specifically tested whether: (1) peer victimization in fall 7th grade would positively predict DPA in fall 8th grade, and then, the DPA would also positively predict NBA in fall 9th grade, and have a direct effect on IGA. (2) NBA would be a key part of a sequential mechanism in the prediction of IGA, which means higher peer victimization will predict higher NBA without the influence of DPA. Taken together, the two mediators of DPA and NBA were expected to shed light on the relationship between peer victimization and IGA.

## Materials and Methods

### Participants

Using random cluster sampling, we recruited the participants from two junior high schools in southern China. Participants completed questionnaires in October of 7th, 8th, and 9th grade. At the T1 baseline assessment (fall 7th grade), 386 seventh graders participated. At T2 (fall 8th grade) and T3 (fall 9th grade), with 351 and 323 of them retained, respectively. No differences were identified on gender, age, and other key variables in the comparison between students who completed all phases of the study and those who dropped out at the second or third assessment.

There were 323 students (52.94% females, *M* age = 14.83, *SD* = 0.49, range = 13.50–16.50) who completed the assessments at all three time points. More than 80% of participants had at least one brother or sister, 65% of their fathers and 76.5% of their mothers had less than high school education. For 82.2% of participants, family income was less than ¥5000 per month.

### Measures

#### Peer Victimization

We assessed peer victimization at T1 with nine questions adapted from the bullying/victim questionnaire (Olweus, 1996; Zhou et al., 2014). The measure has three parts, namely physical aggression, relational aggression, and verbal aggression. The subjects were asked to report the frequency of violations committed by their peers in the past 6 months, including “Have you been beaten, kicked, pushed, or bumped by your peer?” “Have you ever been abused by your peer?” and “Have you been blackmailed, threatened, or intimidated by your peer?” All items are rated on a 5-point scale (from *1 = never* to *5 = four times or more*). We calculated the mean of all items, with higher scores reflecting a greater level of peer victimization. In the current study the Cronbach’s alpha coefficient of this questionnaire was 0.93.

#### Deviant Peer Affiliation

At both T1 and T2, DPA was assessed using a 16-item questionnaires (Elliott and Menard, 1996), which has shown excellent reliability and validity in a large Chinese sample (Jiang et al., 2016). The participants have were asked to report the number of their friends had participated in deviant behaviors (e.g., physical aggression, truancy or absenteeism, stealing, smoking, alcohol use, cheating on exams, vandalism, telling lies, Internet addiction, and gambling) in the past half year on a 5-point scale (from *1 = none* to *5 = six or more*). An example item is “Among your friends, how many people do you think they have stolen?” We calculated the average of the ten items, with higher scores meaning a greater level of DPA. In the current study, Cronbach’s alphas for T1 and T2 assessments were 0.91 and 0.86, respectively.

#### Normative Beliefs About Aggression

At all T1, T2, and T3, the NBA Scale was used to assess beliefs about the general acceptability of aggressive responses (Huesmann and Guerra, 1997). On this 8-item scale, some items directly tap aggressive beliefs, “In general, it is OK to take your anger out on others by using physical force,” and other items indirectly tap aggressive beliefs, for example, “If you’re angry, it is OK to say mean things to other people.” All items were rated on a 5-point scale (from *1 = very disagree* to *5 = very agree*) and the average value was calculated, with higher scores showing more supportive beliefs about aggression. In the present sample, Cronbach’s alphas for T1, T2, and T3 assessments were 0.93, 0.72, and 0.73, respectively.

#### Internet Gaming Addiction

Internet gaming addiction was measured at all three Waves with 16 items adapted from prior published questionnaires (Yu et al., 2015). Adolescents were asked to report the degree of engagement in Internet games during the past half year, for example, “Do you think you would like to spend increasing time in playing online games?” and “Do you go to play online games late at night instead of sleeping?” All items were rated on a 3-point scale (from *1 = never* to *3 = frequently*). Item scores were averaged to create a composite IGA score, with higher scores indicating a higher risk of IGA. In the current sample, Cronbach’s alphas for T1, T2, and T3 assessments were 0.93, 0.83, and 0.87, respectively.

### Procedures

Well-trained psychology graduated students conducted the investigation in class using a standardized data collection process. First, written informed consent was provided by parents and teachers, and assent was provided by the students. The participants received instructions from the trained students and were asked for to complete the questionnaires honestly and independently. They were told that all of their responses and any identifying information would be strictly protected. The entire assessment took approximately 45 min. The questionnaires were collected right after they were finished.

### Data Analysis Approach

First, the inter-correlations of all the variables were computed. Second, we followed a two-step procedure suggested by Anderson and Gerbing (1988) to test our hypothetical model using structural equation modeling in Mplus 7, with the full-information maximum likelihood estimation (Muthén and Muthén, 2013). First, we tested the measurement model to assess the extent to which each of the two latent variables was measured by its indicators respectively. If the fit index of the confirmatory measurement model is satisfactory, further tests of the hypothetical structural model are conducted.

Peer affiliation was measured at both T1 and T2, and normal beliefs about aggression and IGA were assessed at all three waves. All analyses controlled for gender, impulsivity and each variable’s prior wave in order to see if the model still held with baseline controls (Griffiths et al., 2004).

The following three fit indices are recommended by statisticians to evaluate the model’s goodness of fit: (1) the chi-square statistic (χ^2^), χ^2^/*df*, (2) the comparative fit index (CFI), and (3) the root mean square error of approximation (RMSEA). Model fit is acceptable when the χ^2^/*df* ratio is less than 5, CFI value is above 0.95, and the RMSEA value is below 0.08 (Hu and Bentler, 1999). In the current study, all variables were standardized to reduce multi-collinearity. Given that some of the variables were not normally distributed, bias-corrected bootstrap confidence intervals (1000 bootstrap samples) were used to test the statistical significance of the path coefficients (MacKinnon and Luecken, 2008).

## Results

### Descriptive Statistics and Correlation

**Table 1** presents the mean values and standard deviations of all variables, and bivariate correlations among them. As we hypothesized, correlations among the key four variables were significant.

**Table 1:** Descriptive statistics and bivariate correlations between all study variables.

	1	2	3	4	5	6	7	8	9
1. Gender	1.00								
2. Age	0.09	1.00							
3. PV T1	0.00	-0.06	1.00						
4. DPA T1	0.02	0.03	0.41***	1.00					
5. DPA T2	0.11*	-0.06	0.40***	0.42***	1.00				
6. NBA T1	0.13*	-0.05	0.21***	0.13*	0.23***	1.00			
7. NBA T2	0.23***	-0.06	0.24***	0.11*	0.33***	0.38***	1.00		
8. IGA T2	0.34***	0.07	0.15**	0.05	0.28***	0.25***	0.27***	1.00	
9. IGA T3	0.30***	0.03	0.18**	0.14*	0.26***	0.17**	0.40***	0.52***	1.00
*M*	0.53	14.83	1.31	1.22	1.28	1.68	1.82	2.07	1.82
*SD*	0.50	0.49	0.35	0.40	0.57	0.72	0.80	2.19	2.15


*Gender was dummy coded such that 1 = male and 0 = female. PV, peer victimization; DPA, deviant peer affiliation; NBA, normative beliefs about aggression, IGA, internet gaming addiction. ^∗^*p* < 0.05, ^∗∗^*p* < 0.01, ^∗∗∗^*p* < 0.001.*

### Structural Model

**FIGURE 2:**
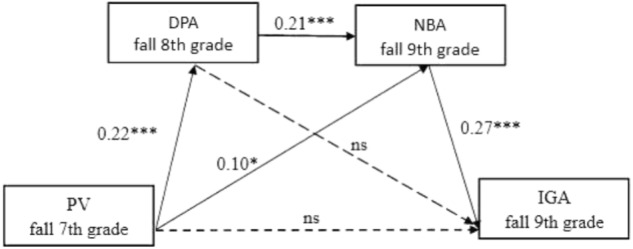
Path model results with standardized coefficients. PV, peer victimization; DPA, deviant peer affiliation; NBA, normative beliefs about aggression; IGA, internet gaming addiction; ns, non-significant. ^∗^*p* < 0.05; ^∗∗∗^*p* < 0.001.

The full mediation model provided an excellent fit with the data: χ^2^ = 15.465, *df* = 11, χ^2^/*df* = 1.406, CFI = 0.990, RMSEA = 0.035. As shown in **Figure 2**, consistent with the hypothesized model, fall 7th grade peer victimization significantly predicted fall 8th grade DPA, which in turn significantly predicted fall 9th grade NBA, which ultimately significantly predicted fall 9th grade IGA. In addition, the another indirect pathway, fall 7th grade peer victimization → 9th grade NBA → 9th grade IGA, was also significant.

## Discussion

The current study goes beyond previous research by using a 2-year longitudinal design to examine the mediating effects of DPA and NBA in the association between peer victimization and the development of adolescent IGA. Consistent with predictions derived from the social network model, ecological systems theory (Bronfenbrenner, 1977), and social learning theory (Bandura and Walters, 1963), this study supported an expansion pathway from peer victimization to DPA to NBA to IGA during adolescence. These findings highlight the significance of peer relations and adolescent cognitions as risk factors for IGA.

Building on previously studies showing that peer victimization positively predicted IGA, findings from our simple mediation model suggest that DPA is one underlying mechanism explaining why victimized adolescents are at risk for IGA. This assumption is congruent with the finding that DPA and NBA were serial mediators of IGA. By affiliating with deviant peers, bullied adolescents are at increased risk of IGA, in part because they see aggression as more normative (Ko et al., 2009). Thus, the results were consistent with our expectations: peer victimization positively predicted DPA, and it in turn was associated with NBA, which positively related to IGA.

Crucially, the results also partially supported our second hypothesis that NBA, as a psychosocial factor, might strengthen the potential pathways explaining how peer victimization has an indirect effect on IGA. Social learning theory emphasizes the robust direct association between peer victimization and NBA. In this model, victimized youths exhibit violence as a way of solving problems and getting needs met (Bandura and Walters, 1963), and may become addicted to the experience of violence in online games (Williams and Guerra, 2007).

There are several reasons why we want to emphasize the particularities of the Chinese context associated in interpreting the results. Firstly, the issues of peer victimization in China are more serious and frequent than in other countries. In fact, it is a large challenge for Chinese school managers to take effective measures to detecting and addressing peer victimization, in part because the very large number of students at any given school. This situation is likely also relevant to detecting and addressing IGA. Secondly, the collectivism of Chinese culture requires individuals to live in various groups, and the influence of DPA is a factor that cannot be ignored. It is easy to understand why victimized adolescents might choose a group of deviant peers as friend when they are not able to join the main stream group; being in any group is better than being alone. The cultural context in which gaming takes place is completely in conformity with the individual’s needs for the collective, which embeds the gamer in a community with shared beliefs and practices, endowing their gaming with particular meaning. Thirdly, the macro-system, namely China as a modesty culture, is one that holds the normative belief that aggression is to be avoided. As the old Chinese saying goes, “we should convince people by reason, but not resort to force.” However, our results have supported that a negative peer context does affect adolescents’ NBA, and further influences IGA. To sum up, the findings are informative for further research to determine if there is cultural variation in the risk for adolescent IGA.

The results are important because, despite the extensive body of research on IGA, few studies have taken into account multiple predictors or assessed other than short-term effects. To our knowledge, our study is one of the first to use a theoretical perspective to study adolescent IGA, and to use 2-year longitudinal data. By expanding on previous studies, this research provides a novel perspective on how peer victimization can lead to adolescent IGA through its effects of DPA and NBA. Findings from this study suggest that reducing NBA may have a protective effect for adolescents at risk of IGA. Future research should examine processes that foster resilience in this vulnerable population.

## Conclusion, Limitations, and Future Directions

Overall, this research makes a vital contribution to the field of IGA by identifying DPA and NBA as factors that link peer victimization to adolescent IGA. Nevertheless, several limitations should be noted. First, data were collected in the fall of each year, and it is possible that students who were exposed to victimization in the fall would no longer report such experiences in the spring. Second, the participants were about 15 years of age at the last assessment (in 9th grade). Future research ought to extend the sample age, which could allow for greater understanding of these processes in later adolescence. Third, all of our measures were self-reports. The measure of IGA may be particularly troublesome as the participants may underestimate or misrepresent the true level of IGA. Therefore, we should try to use more reliable methods and measures from diversified sources. Fourth, the measure of NBA included very few items, and further scale development may be needed. Fifth, we did not measure violent Internet gaming in particular, instead focusing on the wide range type of online games. Finally, it is possible that NBA will be influenced by IGA in turn. Future research would benefit by examining the bidirectional effects in the associations among beliefs, peer relations, and IGA.

Despite these limitations, our study suggests critical points of intervention. For example, adolescents exposed to peer victimization may develop feelings of anger (Kochenderfer-Ladd, 2004) that reinforce NBA and push them to affiliate with deviant peers, increasing their risk to become addicted to Internet gaming. Helping victimized teenagers to stay engaged in mainstream instead of deviant peer groups, and to avoid NBA, may be keys to preventing IGA. These findings demonstrated the significance of serial mediation models in understanding the mechanisms by which peer victimization is associated with adolescent IGA.

## Ethics Statement

This study was carried out in accordance with the recommendations of the Research Ethics Committee at South China Normal University guidelines, the Research Ethics Committee at South China Normal University with written informed consent from all subjects. All subjects gave written informed consent in accordance with the Declaration of Helsinki. The protocol was approved by the Research Ethics Committee at South China Normal University.

## Author Contributions

As the first author, PS participated in design of this study, collected data, performed the statistical analysis, and completed the manuscript. WZ, a respectable, responsible and resourceful scholar, who has provided PS with valuable guidance in every stage of the writing of this paper. His keen and vigorous academic observation enlightens me not only in this thesis but also in my future study. CY has given his constant help for data analysis, and read the manuscript with great care and offered me invaluable advice in design part. SL and SZ also have helped me to develop the fundamental and essential academic competence. YX as a good teammate, joined the data collection and gave me lots of informative suggestions within the writing.

## Conflict of Interest Statement

The authors declare that the research was conducted in the absence of any commercial or financial relationships that could be construed as a potential conflict of interest.
